# Genotyping Single Nucleotide Polymorphisms Using Different Molecular Beacon Multiplexed within a Suspended Core Optical Fiber

**DOI:** 10.3390/s140814488

**Published:** 2014-08-08

**Authors:** Linh Viet Nguyen, Sara Giannetti, Stephen Warren-Smith, Alan Cooper, Stefano Selleri, Annamaria Cucinotta, Tanya Monro

**Affiliations:** 1 Institute for Photonics and Advanced Sensing (IPAS) and The ARC Centre for Nanoscale Biophotonics, The University of Adelaide, Adelaide 5005, Australia; E-Mails: stephen.warrensmith@adelaide.edu.au (S.W.-S.); tanya.monro@adelaide.edu.au (T.M.); 2 Information Engineering Department, University of Parma, Parma 43100, Italy; E-Mails: s.giannetti87@gmail.com (S.G.); stefano.selleri@unipr.it (S.S.); annamaria.cucinotta@unipr.it (A.C.); 3 Australian Centre for Ancient DNA (ACAD), The University of Adelaide, Adelaide 5005, Australia; E-Mail: alan.cooper@adelaide.edu.au

**Keywords:** genotyping, suspended core optical fiber, molecular beacon, multiplexing, DNA detection

## Abstract

We report a novel approach to genotyping single nucleotide polymorphisms (SNPs) using molecular beacons in conjunction with a suspended core optical fiber (SCF). Target DNA sequences corresponding to the wild- or mutant-type have been accurately recognized by immobilizing two different molecular beacons on the core of a SCF. The two molecular beacons differ by one base in the loop-probe and utilize different fluorescent indicators. Single-color fluorescence enhancement was obtained when the immobilized SCFs were filled with a solution containing either wild-type or mutant-type sequence (homozygous sample), while filling the immobilized SCF with solution containing both wild- and mutant-type sequences resulted in dual-color fluorescence enhancement, indicating a heterozygous sample. The genotyping was realized amplification-free and with ultra low-volume for the required DNA solution (nano-liter). This is, to our knowledge, the first genotyping device based on the combination of optical fiber and molecular beacons.

## 1. Introduction

Single-nucleotide substitutions represent the largest source of diversity in the human genome. Although the vast majority are neutral, these variations have also been directly linked to human disease [1]. Even neutral variations are important because they provide markers for the preparation of detailed maps of the human genome, serving as essential elements in linkage analyses that identify genes responsible for complex disorders [2]. Although sequencing is adequate for the initial discovery of single-nucleotide variations, simpler, faster and more automated genotyping methods are needed to understand the distribution of genetic variations in populations, as well as for identifying the genes responsible for genetic disorders [1]. There have been various protocols and methods proposed for genotyping single-nucleotide polymorphisms (SNPs). For example, methods that utilize gel electrophoresis to identify single or double-stranded DNA polymorphisms [3,4]). SNPs can also be determined using solid-phase chemical cleavage [5] in which commercially available chemicals are used to modify cytosine and thymine, respectively. The modification of the mismatch is then followed by cleavage with piperidine and the resulting DNA fragments are analyzed by denaturing polyacrylamide gel-electrophoresis to identify the mismatch sites [1]. SNPs genotyping can as well be carried out through DNA sequencing, in liquid phase [6] or solid-phase [7,8] such as using oligonucleotide microarrays [8]. In addition 5′-nuclease reaction [9], or mass spectroscopy [10] have all been utilized for SNPs genotyping. A comprehensive list of typical SNP genotyping methods and their details can be found in Reference [1]. Despite their sophistication, typically the SNP genotyping techniques mentioned above requires either polymerase chain reaction (PCR) or electrophoresis or both.

Molecular beacons (MBs) are single-stranded oligonucleotide hybridization probes that form a stem-and-loop structure [11]. The loop contains a probe sequence that is complementary to a target sequence, and the stem is formed by the annealing of complementary arm sequences located on either side of the probe sequence. A fluorophore is covalently linked to the end of one arm and a quencher is covalently linked to the end of the other arm. In the absence of target DNA, the probe is dark because the stem places the fluorophore so close to the quencher that they transiently share electrons and the fluorescence is efficiently quenched. When the probe encounters a target molecule it forms a probe-target hybrid, which is longer and more stable than the stem hybrid. Consequently, the molecular beacon undergoes a spontaneous conformational reorganization that forces the stem hybrid to dissociate and the fluorophore and the quencher to move away from each other, restoring fluorescence [11]. MBs are well known to be highly specific and capable of real-time monitoring of DNA amplification during a polymerase chain reaction [11] and are thus widely used as a probe for DNA detection in various applications [12], including SNP genotyping [1]. With its capability to recognize the target sequence in a pool of many different sequences and thus eliminating the process of sorting out DNA segments by electrophoresis, attaching MBs on an ultra sensitive transduction platform in which a very low concentration of target sequence can be detected will lead to a much simpler SNP genotyping device.

Over the last two decades, many approaches have been explored for the use of optical fibers in biochemical sensing applications, including DNA sensing [13]. Since optical fibers developed for telecommunications applications were designed to well isolate the light propagating in the core from the ambient environment, many advanced designs of optical fiber or fiber-optic devices have therefore emerged to facilitate the interaction of guided light with the target biochemical analytes. Among such optical fiber designs for biochemical sensing purposes, the development of a special type of optical fiber named “suspended core microstructured optical fiber”, which can provide strong interactions between the guided light in the fiber core and samples loaded within the fiber voids in addition to simple filling characteristics, while being simple to fabricate has been proposed and developed in our group [14]. The SCF has been demonstrated for a variety of biochemical sensing applications based on fluorescence measurements such as selective detection of biomolecules [15], chemicals [16], and real-time distributed measurements using exposed-core SCF [17] or specific DNA sensing in a dip-sensing fashion [18]. SCFs are hollow fibers with a small (micron-scale) solid core supported by a few thin struts (3 or more struts depending on the design) reflecting the name “suspended core fiber”. By fabricating the fiber such that it has a core that is comparable to or smaller than the wavelength of light guided in the fiber, the portion of the light guided by the fiber that is located within the air voids can be significantly enhanced. Solutions under examination can be loaded into the air holes of the SCFs for direct interaction with this portion of the guided light, leading to the potential for high sensitivity [14]. Measurement sensitivity for SCF based detection is typically ranging from nano- to pico-molar or even down to the level of single particle sensitivity [19]. Finally, since the dimension of the air voids is also of micrometer scale, the amount of the solution required to fill the void can be very small, in the order of nano-liters, depending on the length of the SCF used for sensing.

In this paper we propose and demonstrate the genotyping of SNPs using molecular beacons multiplexed within an SCF. Typically in the dip-sensing SCF based detection, the SCF itself serves in an all-in-one fashion as the substrate for biological immobilization, fluorescence excitation and collection platform, sample handling tip (liquid under test can be “sucked” into the void micro-scaled air-holes through capillary force simply by dipping the fiber tip to the sample), thus reducing the operation and measurement complexity. In addition since MBs are sophisticated DNA probes, they are typically synthesized with relatively low yield compared to their linear DNA probe counterpart and thus have a high cost, particularly when a large amount of material is required for detection and/or analysis. In this aspect, the low-volume sensing capability of SCF as a genotyping platform is advantageous, as it would greatly allow reduction of the material cost in sensor fabrication as well as during hybridization. Last but not least, the large surface-to-volume ratio nature of the SCF platform leads to rapid heat dissipation. For natural genome DNA it is always necessary to denature the doubled stranded DNA as well as amplify selected loci along the entire DNA, typically with PCR, before detection takes place. The thermal-efficient characteristic of SCF, in conjunction with its ultra low volume sensing, will allow rapid heating/cooling, and thus fast PCR to be realized.

## 2. Experimental Section

### 2.1. Immobilization of Dual-Type MBs on the Surface of the SCF Core

**Figure 1.**
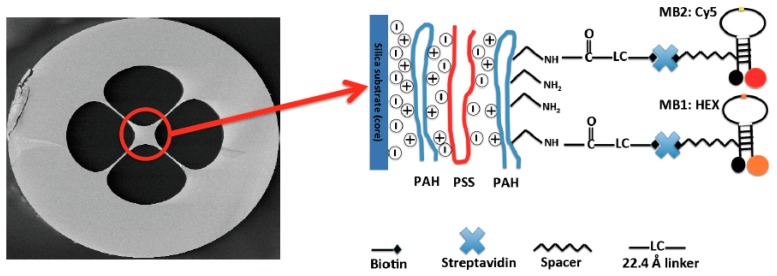
Schematic diagram of the final stage of the suspended core optical fiber (SCF) core functionalized with dual-type molecular beacons. Picture of the SCF cross section shown on the right side is a scanning electron microscope (SEM) image of the SCF used in this work.

The immobilization of two types of MBs on the surface of the SCF core was carried out following the procedure detailed in our previous publication [18] using a combination of the fuzzy nanoassembly technique [20] and the biotin-streptavidin binding mechanism [21]. An in-house made pump system was used to flow the required solutions through the SCF air-holes for deposition of different immobilization layers. Typically one end of SCF was immersed in solution vial placed in a sealed chamber and the other end was left free outside of the chamber. A pressure difference between the inside and outside of the sealed chamber was created by a pump connecting to the chamber, forcing the liquid to move from inside to outside through the SCF air-holes. Since the SCF surface is partly negatively changed by OH^−^ groups on the surface, positively charged poly(allylamine) hydrochloride (PAH, 2 mg/mL in 1 M NaCl solution, Sigma Aldrich) and negatively charged poly(sodium 4-styrene sulfonate) (PSS, 2 mg/mL in 1 M NaCl solution, Sigma Aldrich) were deposited alternately onto the fiber core surface using the layer by layer deposition technique described in [20], ending with a PAH layer (PAH/PSS/PAH). The PAH provides amino groups for immobilization of biotinylated MB through a biotin-streptavidin-biotinylated MB link [21]. The flow time for each polyeletrolyte layer was 20 min, followed by extensive rinsing using deionized (DI) water. NHS-LC-Biotin (0.5 mg/mL, Thermo Fisher) was prepared freshly and flowed through for 1 h, followed by extensive rinsing using phosphate buffer solution (PBS, Sigma Aldrich) to remove unbound biotin on the surface. Non-specific blocking solution (Candor) was flowed through the fiber for 2 h and rinsed by PBS. Streptavidin (0.5 mg/mL, Thermo Fisher) was flowed though for 40 min at room temperature then left inside the air-holes overnight and then rinsed thoroughly with PBS. In this case Streptavidin only binds to Biotin rather than non-specifically deposition on the SCF surface thanks to the use of non-specific blocker. A biotinylated MB solution containing a mixture of two different MBs (2.5 μM for each MB, Midland Certified Reagent Company Inc.) was flowed through for another 1 h, rinsed with PBS and DI water and then dried with Nitrogen for 15 min. The functionalized fiber was cut into several pieces of 65 mm length each for measurement. Figure 1 shows the cross section of the SCF used in this work and a sketch of the final state of the SCF core surface after immobilization. The SCF was a silica glass SCF made in-house with a core diameter of approximately 13 μm and has four air-holes. We chose to use a relatively large core SCF principally to ensure high optical coupling stability during the fluorescence measurement. Despite the large core diameter, we found that the dual-type MB immobilized SCF presented in this work can detect DNA solution whose concentration of as low as 100 nM. If one desires to increase further the sensitivity of the sensor, a smaller core size should be used, however, at the expense of coupling instability. In this case, advanced fiber processing techniques such as well-optimized fusion splicing are required to ensure good optical coupling stability [22,23].

### 2.2. Measurement Setup

**Figure 2.**
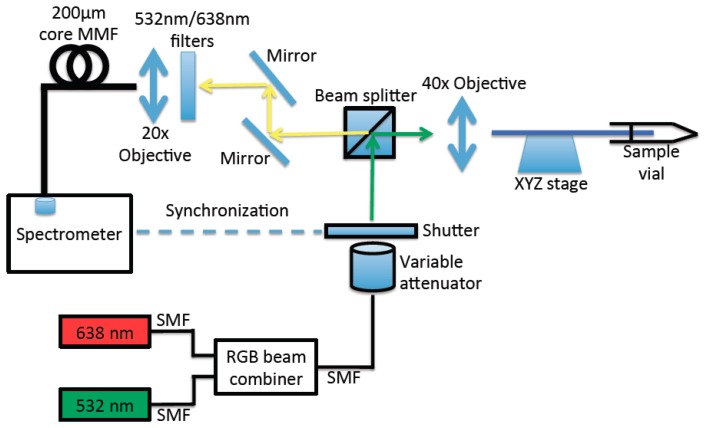
Experiment setup for fluorescence measurement of the dual-type molecular beacons (MBs) immobilized SCF filled with DNA solutions. SMF and MMF are abbreviations for single mode and multimode mode optical fiber, respectively. The green laser operating at 532 nm was used to excite HEX dyes (for wild-type sequence) and the red laser operating at 638 nm was used to excite Cy5 dyes (for mutant-type sequence).

A schematic diagram of the measurement setup for fluorescence measurement of the dual-MB immobilized SCF is shown in Figure 2. Two lasers operating at two excitation wavelengths corresponding to HEX and Cy5 dyes have been used. Excitation lights from a 532 nm laser (Crystal Laser) and a 638 nm laser (Toptica) were first combined by a RGB fiber beam combiner (Thorlabs), power-adjusted by a variable attenuator (a pair of half-wave plate and polarizer), and then directed to the SCF using a beam splitter and a 40× microscope objective. The same microscope objective and beam splitter also serves as the collecting objective for the backscattered fluorescence from the fiber. Backscattered light was directed through a system including mirrors, a filter (either 532 nm or 638 nm filter depending on what MB signal is being interrogated) to block the residual pump light, and coupled to a large core multimode fiber using a 20× microscope objective. The multimode fiber guides the backscattered light to a spectrometer. A shutter that was synchronized with the spectrometer was placed after the output of the beam combiner. The consistent mode coupling in each measurement was achieved by means of maximizing the power transmitted through the SCF core. It should be noted that Cy5 dye is known to have photoswitching characteristic [24], *i.e.*, exposure of Cy5 under continuous wave laser light at 638 nm turns the dye into a dark state, which then can be switch back to a fluorescent state with 532 nm exposure [25]. Therefore when maximizing light coupling to the SCF core, the green laser (532 nm) was mainly used, followed by a weak red light (638 nm) beam to check the power coupling to the SCF core for the red wavelength. The continuous red light exposure of the immobilized SCF was typically 3 s to minimize the potential photoswitching effect on the Cy5. In the measurement of fluorescence enhancement upon filling the immobilized SCF with DNA solution, the green laser was always used first. In this way the green light was used both to excite the fluorescence from HEX dye as well to reactivate the Cy5 dye to a fluorescent state, in case it might have been photoswitched to a dark state, even by the weak red beam.

### 2.3. Hybridization Experiment

The hybridization test between the dual-type MB immobilized SCF and the solution containing either homozygous single stranded DNA (wild or mutant) or both types (presenting a heterozygous mixture) were carried out in a manufacturer-recommended buffer solution containing 20 mM Tris-HCl, 5 mM MgCl2, and 50 mM KCl, at room temperature (25 °C). The background of each piece of fiber under test was recorded first and all the fluorescence spectra were normalized with their own backgrounds to extract the fluorescence enhancements. Not all of the fiber pieces showed a proper fluorescence background; some of the pieces cut from the same functionalized SCF exhibited a very weak background, indicating that the immobilization along a long fiber might not be ideally uniform. Therefore those poorly functionalized fiber pieces were dismissed and only the pieces with clear and similar fluorescence background at HEX and Cy5 wavelengths were used for measurement. The filling time for all the fiber pieces was approximately 30 s. After filling, fluorescence from HEX dye (associated with wild-type MB) was collected first, followed by changing the filter and switching the laser beam to excite and collect the fluorescence from Cy5 (associated with mutant-type MB). The MBs and DNA sequences used in this work are given in Table 1. For the purpose of demonstrating the proof-of-concept, in this work the wild and mutant MBs and the corresponding DNA sequences were arbitrarily designed by the manufacturer with a quoted discriminating capability, between the target and the one-base mismatching sequence by a factor of 10, as measured in solution. However, virtually any sequences of practical interest could be detected by immobilizing the correspondingly designed MBs on the SFC core.

## 3. Results and Discussion

### 3.1. Verification of the Immobilization Process with Control SCF

**Figure 3.**
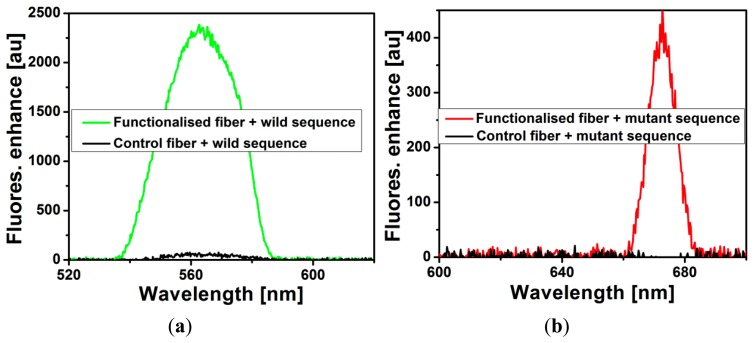
Comparison of fluorescence enhancement between dual-type MB immobilized SCF and control SCF upon filling with target DNA solution. The control SCF shows negligible fluorescence enhancement.

Another SCF that serves as the control fiber was put through the same immobilization process as described in Section 2, except that the biotin-streptavidin linking step was omitted. The two fibers, dual-type MB immobilized fiber and the control fiber, were loaded with solution containing either wild-type or mutant-type sequence with a concentration of 100 nM and the fluorescence were measured. In this work the functionalized was not optimized for any specific concentration as the functionalization involved multiple coating layers which makes optimizing the probe surface density difficult. In principle, detection in a biosensor is based on the binding between functionalized probe (molecular beacon in our case) and analyte suspended in the solution under test and therefore there should be an optimized pair of probe density on the sensor surface and analyte concentration. As can be seen in Figure 3, it is clear that the two MBs are successfully immobilized on the surface of the SCF core through the biotin-streptavidin link as the fluorescence of the MB immobilized fiber increased significantly upon hybridizing with target DNA while that of the control fiber remains approximately unchanged. This indicates that without the intended biotin-streptavidin link, biotinylated MBs cannot form a stable chemical attachment to the fiber surface and are removed when rinsed. The use of the non-specific blocking layer helps to ensure that biotinylated MB binds only to the surface through the biotin-streptavidin link and not directly to the surface due to physical adsorption, which would be too close to the surface and thus might be associated with high steric hindrance.

### 3.2. Genotyping SNP with Dual-Type MB Functionalized SCF

**Figure 4.**
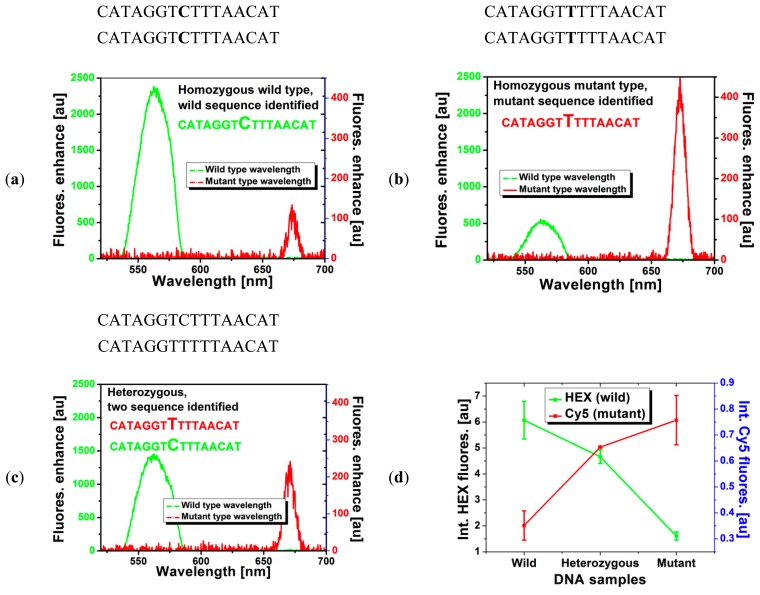
Hybridization test of the dual-type MB immobilized SCF filled with solution containing either one type of DNA sequence, e.g., wild or mutant sequence (homozygous) or both type (heterozygous). Fluorescence enhancement clearly indicates (**a**,**b**) the homozygous type or (**c**) heterozygous type; (**d**) The averaged value over four measurements of spectra as shown in (a–c), crossing point of HEX and Cy5 fluorescence indicate heterozygousity and well separated values of HEX and Cy5 fluorescence represent homozygousity.

The results of the hybridization test, in which the dual-MB immobilized SCFs were loaded with different DNA solution containing either one type of DNA or both and fluorescence enhancements were recorded, is shown in Figure 4. When the fiber was filled with a buffer solution containing one type of DNA, either wild-type or mutant type (Figure 4a,b), essentially only one type of MB was undergoing conformational change due to target sequence binding to the probe in the MB loop, as evidenced by the enhancement of fluorescence at only one wavelength (Figure 4a is for fiber filled with wild-type DNA solution and Figure 4c is for fiber filled with mutant-type DNA solution). On the other hand, once the fiber is filled with DNA solution containing both the wild and mutant sequence (Figure 4c), both MBs experienced conformational changes as the fluorescence enhancement were obtained at both wavelengths. Of course, the level of enhancement should be lower than the case of single DNA sequence since it is a competing reaction, consistent with the case of genotyping SNPs using MBs in solution [1]. Therefore, the proposed dual-type MB immobilized SCF clearly functions as a SNP genotyping platform. Figure 4d shows the integrated intensity for the HEX (left) and Cy5 (right) fluorescence. Here sample is considered heterozygous if the two integrated intensities of the two dyes are close to each other while well-separated values of the fluorescence indicate homozygosity. It should be noted that the performance of the MBs immobilized on the surface of the SCF was somewhat deteriorated compared with the case of in-solution measurement. This is partly due to the steric hindrance of the surface on the MB as well as MBs to each other. In general it is not possible to obtain similar performance for in-solution (same phase) reaction and immobilized/liquid (different phase) reaction however it might be improved by optimizing the buffer condition as well as fine-tuning the design of the MBs.

It should be noted that all hybridization and measurement procedures were performed at room temperature. For the purpose of simplifying this proof-of-concept experiment as well as limit of equipment temperature control was not included in this work. While it is important to have strict temperature control of the functionalized SCF for highly specific allele discrimination in the case of real-world genome DNA samples (in such case the buffer should contain many different DNA sequences extracted from the genome DNA), it is known that MB can still achieve good discrimination between sequences of only one base difference [11], even at room temperature. With proper temperature control and performing the allele discrimination at optimized temperature for a specific pair of MB and target, as well as optimizing buffer and MB design, the discrimination should be greatly enhanced. In addition, for genome DNA temperature control is often essential, not only for enhancing the allele discrimination but also for performing other tasks such as denaturing the double stranded genome DNA as well as amplifying selected loci on the entire sequence before detection. Finally, with temperature control on the functionalized SCF, the sensor can in principle be regenerated by denaturing the DNA-probe binding using heat and flowing fresh buffer through the air-holes. DNA-probe binding in MB is well known to be reversible, as evidence by its usage in real-time PCR [26], and therefore as long as the DNA is removed MB should resume its original stem-loop and regenerate the sensor.

## 4. Conclusions/Outlook

We have presented the first demonstration of the use of SCF in conjunction with MBs for genotyping SNPs. The proposed device is based on the functionalization of multiple MBs on the core of an SCF and is capable of genotyping SNPs in a DNA solution whose concentration is as low as 100 nM. The sensing volume is in the nano-liter range and the genotyping protocol can be as simple as dipping the free end of the functionalized SCF into the solution under test, leading to potentially highly accurate and cost-effective genotyping SNPs protocol. Further work is expected to increase the sensitivity of the device by reducing the SCF core size, optimizing the coupling as well as the MB designs and buffer conditions. By further driving the sensitivity of the sensor down to the level of a few, or even a single, DNA template, the proposed genotyping scheme should be able to perform the genotyping of SNPs in a simpler manner, without the need for PCR or electrophoresis.

## Figures and Tables

**Table 1.** Molecular beacons and DNA sequences used for testing the immobilized SCF. Samples were synthesized by the Midland Certified Reagent Company Inc. Concentration of DNA solution used in the hybridization experiment is 100 nM.

**Molecular Beacon**

Wild-type MB:
5′-(HEX)AGCGGATGTTAAA**G**ACCTATGCCGC(BHQ1-dT)(spacer 18)(3′-Biotin)-3′
Mutant-type MB:
5′-(HEX)AGCGGATGTTAAAA**A**CCTATGCCGC(BHQ1-dT)(spacer 18)(3′-Biotin)-3′

**DNA Sequences**

Wild-type sequence: 5′-CATAGGT**C**TTTAACAT-3′
Mutant-type sequence: 5′-CATAGGT**T**TTTAACAT-3′
